# Acupuncture in the treatment of rheumatoid arthritis: a double-blind controlled pilot study

**DOI:** 10.1186/1472-6882-7-35

**Published:** 2007-11-03

**Authors:** Lai-Shan Tam, Ping-Chung Leung, Tena K Li, Lang Zhang, Edmund K Li

**Affiliations:** 1 The Department of Medicine & Therapeutics, The Prince of Wales Hospital, The Chinese University of Hong Kong, Hong Kong, China; 2 The Institute of Chinese Medicine, The Prince of Wales Hospital, The Chinese University of Hong Kong, Hong Kong, China

## Abstract

**Background:**

In planning a randomized controlled trial of acupuncture, we conducted a pilot study using validated outcome measures to assess the feasibility of the protocol, and to obtain preliminary data on efficacy and tolerability of 3 different forms of acupuncture treatment as an adjunct for the treatment of chronic pain in patients with Rheumatoid arthritis (RA).

**Methods:**

The study employs a randomized, prospective, double-blind, placebo-controlled trial to evaluate the effect of electroacupuncture (EA), traditional Chinese acupuncture (TCA) and sham acupuncture (Sham) in patients with RA. All patients received 20 sessions over a period of 10 weeks. Six acupuncture points were chosen. Primary outcome is the changes in the pain score. Secondary outcomes included the changes in the ACR core disease measures, DAS 28 score and the number of patients who achieved ACR 20 at week 10.

**Results:**

From 80 eligible patients, 36 patients with mean age of 58 ± 10 years and disease duration of 9.3 ± 6.4 years were recruited. Twelve patients were randomized to each group. Twelve, 10 and 7 patients from the EA, TCA and Sham group respectively completed the study at 20 weeks (p < 0.03); all except one of the premature dropouts were due to lack of efficacy. At week 10, the pain score remained unchanged in all 3 groups. The number of tender joints was significantly reduced for the EA and TCA groups. Physician's global score was significantly reduced for the EA group and patient's global score was significantly reduced for the TCA group. All the outcomes except patient's global score remained unchanged in the Sham group.

**Conclusion:**

This pilot study has allowed a number of recommendations to be made to facilitate the design of a large-scale trial, which in turn will help to clarify the existing evidence base on acupuncture for RA.

**Trial registration:**

ClinicalTrials.gov NCT00404443

## Background

In recent years, patients with chronic rheumatic disorders are adopting complementary/alternative medicine (CAM) to help manage their chronic painful conditions. In previous reported arthritic studies, the prevalence of CAM was between the range of 33–90% [1]. Collectively the evidence demonstrates that some CAM modalities show significant promise such as acupuncture, diets, herbal medicine, homoeopathy, massage and supplements. Of these modalities, acupuncture has been shown to be effective for a number of painful conditions including chronic knee [2] and neck pain [3]. It is defined as the stimulation of a certain point or points on the body, by the insertion of needles, to achieve a desirable effect. It is thought to prevent or modify the perception of pain or to alter physiologic functions, including pain control for the treatment of certain diseases or dysfunctions of the body [4]. Acupuncture typically includes manual stimulation of needles, but there are variations commonly used, such as electrical stimulation and heat stimulation of needles, which is called moxibustion: the moxa herb, *Artemisia vulgaris*, is burned at the handle end of the needle. Injection acupuncture, in which herbal extracts are injected into acupuncture points, are occasionally used as well [5]. It is unclear which type of acupuncture produces the most beneficial effect [6].

Recent randomized trials and meta-analysis in patients with osteoarthritis of the knee, acupuncture has been shown to provide pain relief and improvement in joint function [7-9]. In contrast, evidence for this form of treatment in inflammatory joint diseases is sparse and is inconclusive [10,11]. The effect of traditional Chinese acupuncture (TCA) or electroacupuncture (EA) on the objective and subjective measures of disease activity in patients with RA was reviewed recently [12]. There were only 2 studies which were suited for analysis. In one cross-over study, acupuncture was considered ineffective [11]. Questions were raised in the methodology including the choice of the acupuncture point, the treatment duration and frequency [13]. The second study compared electro-acupuncture to sham acupuncture. Problems with this study include the purpose for the use hydrocortisone injection which may have impacted on the results. Other concerns were the outcome measures used as well as the methodology of reporting the results [12].

In planning a randomized controlled trial of acupuncture, we conducted a pilot study using validated outcome measures to explore the feasibility of the protocol and obtain preliminary data on efficacy and tolerability of 3 different forms of acupuncture treatment as an adjunct for the treatment of chronic pain in patients with RA. We wished to address the questions of a) the accrual and dropout rates, b) compliance with the protocol, c) adverse event. We also wished to explore d) the feasibility of the planned standardized outcome measures, and e) the variability in the primary clinical outcome measure. We chose these treatments to investigate a specific effect (if any) of the stimulation, or depth of needling (TCA or EA vs. sham acupuncture).

## Methods

### Patient selection

This was a randomized, double-blind, placebo controlled trial. Participants were recruited to the study from the Rheumatology clinic at the Prince of Wales Hospital. The study was conducted at The Institute of Chinese Medicine at The Chinese University of Hong Kong. All patients fulfilled The American College of Rheumatology (ACR) [14] criteria of RA and with active disease affecting the hands and wrists defined as having at least 4 tender joints and 2 swollen joints; early morning stiffness of greater than 45 minutes; ESR > 28 mmHg or CRP > 10. Patients taking disease modifying anti-rheumatic drugs (DMARDs) were eligible if they were on a stable dose for at least 3 months before screening. Patients on stable doses of one non-steroidal anti-inflammatory drug (NSAID) or up to 10 mg daily prednisone were also included. Analgesia, including NSAID, steroids and DMARDs were continued. All patients were instructed not to make any changes in their background therapies during the study. Intra-articular or pulse steroid were not permitted during the study. Exclusions were those who were under the age of 18 years, pregnancy, previously had acupuncture, localized skin infections, anticoagulated and those with bleeding diathesis, intra-articular corticosteroid within 4 weeks preceding the study, or with any severe chronic or uncontrolled co-morbid disease, or fear of needles. Ethical approval was obtained from the Ethics Committee at The Chinese University of Hong Kong. All patients gave written and informed consent at the time of enrolment.

### Randomization and blinding

Thirty-six patients at the Prince of Wales Hospital were recruited and were randomized according to the computer generated randomly allocated treatment codes made by the secretary of the Institute of Chinese Medicine who had no other role in this study. The randomization code used was concealed from investigators (EL and LST) and patients throughout the study.

### Acupuncture treatment

**Figure 1:**
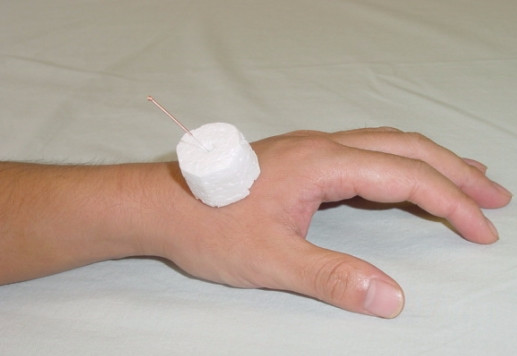
A special device using a 2 cm cube of foam material adherent to the skin around the acupuncture point for mounting the acupuncture needle. The recipient would not be able to see the level of entry of the needle since the tip of the needle was hidden by the adherent cylinder of foam.

All patients received treatment by the registered acupuncturist (LZ) who has been practicing acupuncture for more than 10 years. All patients received two 40 minute – sessions weekly for a total of 20 sessions over a 10 week period. Sterile, (0.25 mm × 25 mm and 0.25 mm × 40 mm), disposable needles were used in all patients. A special device was used for 3 groups of subjects – the acupuncture needle was mounted through a standard 2 cm cube of foam material adherent to the skin around the acupuncture point (Figure 1) [15,16]. The needles were left in place for 30 minutes.

All patients were randomized to one of the 3 treatment groups. Electroacupuncture (EA) Group: Electroacupuncture with needles stimulated by an electrical current (dense wave at 4 HZ and disperse wave at 20 H**Z**) generated by the generator (Model G6805-2) and intermittent non-specific manual twirling. The electrical stimulation of the acupuncture point were started 10 minutes after needle insertion and stopped just before needle withdrawal. Traditional Chinese acupuncture (TCA) group: Acupuncture without any current passing through but receive intermittent specific manual twirling. Sham group: Sham acupuncture. The skin was punctured to a depth of 2 mm and then the needle was quickly withdrawn. The recipient could not see the level of entry since the cube of foam hid the tip of the needles. The needles were connected to the electrical current generator without any current passing through. Intermittent non-specific manual twirling was also applied.

**Figure 2:**
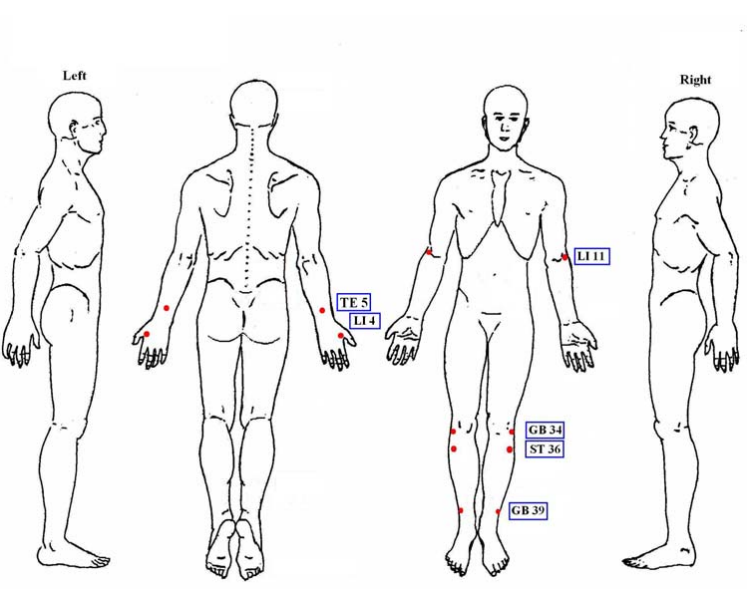
The anatomical location of the six acupuncture points.

Six acupuncture points including Quchi (LI11), Waiguan (TE5), Hegu (LI4), Zusanli (ST36), Yanglingquan (GB34), Xuanzhong (GB39) were used in all patients (Figure 2). Selection of acupuncture points were based on Traditional Chinese Medicine theory of 'Bi' syndrome, which uses local and distal points on channels that traverse the area of pain. Distal points are chosen for the treatment of the underlying causes of Bi syndrome and local acupuncture points were selected for the treatment of the nearby joints. All the 6 acupuncture points chosen can serve as both distal and local points since RA is a systemic disease affecting multiple joints [17]. The anatomical location of the acupuncture points was depicted in Figure 2.

The acupuncturist inserted the acupuncture needles (0.25 mm × 40 mm) for acupuncture points LI11, TE5, ST36, GB36, GB39 to a depth of 20 mm. For acupuncture point LI4, the acupuncturist inserted 0.25 mm × 25 mm acupuncture needles to a depth of 10 mm. Patients in both EA and TCA groups experienced a sense of heaviness, soreness, or numbness at the point of needling called "de Qi". This sensation was said to be a sign that an acupuncture point has been correctly stimulated. The acupuncturist has the least possible communication with patients, and patients were discouraged to communicate with each other before and after the treatment sessions to minimize bias.

### Clinical and laboratory assessment

Assessments at baseline, 5, 10, 15, 20 weeks included clinical and laboratory assessment. Clinical assessment included visual analogue scale (VAS) for pain (0 indicating no pain and 10 indicating worst pain imaginable), the number of swollen and tender joints, patient's global assessment, a validated version of the Chinese Health Assessment Questionnaire (HAQ)[18] and the physician's global assessment which was done by a blinded physician (EL or LST) who performed all examinations and assessments throughout the entire study period.

Laboratory assays including complete blood count, blood chemistry, urinalysis, and liver function tests, erythrocyte sedimentation rate (ESR) and C-reactive protein (CRP) were performed at each visit and potential adverse events were assessed by using open-ended questions at each study visit.

### Outcomes

Primary outcome is the changes in the pain score at week 10. Secondary outcomes included the changes in the ACR core disease measures, including the number of tender and swollen joints, physician global assessment, patient global assessment and the HAQ; DAS 28 score and the number of patients who achieved ACR 20 at week 10.

### Statistical Analysis

The intent-to-treat population was the primary population for efficacy and safety analyses, and comprised all patients who received at least one session of acupuncture. A last-observation-carried-forward approach was used to impute missing data in the intent-to-treat population analysis. Comparisons between the 3 treatment groups for demographic and clinical characteristics were performed using descriptive statistics (Chi-squared tests) and one way ANOVA as appropriate. Comparisons before and after treatment in each group was assessed using paired-t tests or Wilcoxon Signed Rank tests when appropriate. All hypotheses were 2-tailed, and p-values less than 0.05 were considered significant. Analyses were performed using The Statistics Package for Social Sciences (SPSS for Windows, version 13.0, 2006, SPSS Inc, Chicago, IL).

## Results

### Recruitment rate and baseline characteristics

Of the 491 RA patients currently being followed at the Prince of Wales Hospital, 80 were identified between June 2005 to May 2006 who fulfilled the inclusion and exclusion criteria. Of those eligible patients, 36 patients were randomized. Most patients who refused to participate were either not interested, or unable to take time off work. The demographics (including gender differences) and the clinical characteristics of the patients in each group at baseline were similar (Table 1 and 2).

**Table 1:** Demographics and current medications of patients with RA

	Electro-Acupuncture (EA) (N = 12)	Traditional Acupuncture (TCA) (N = 12)	Sham Acupuncture (Sham) (N = 12)
Age, mean ± SD years	56.4 ± 8.5	58.1 ± 12.0	57.6 ± 8.3
Disease duration, mean ± SD years	8.4 ± 5.6	10.8 ± 6.2	8.1 ± 6.9
Male: Female	1:11.	2:10	4:8
Rheumatoid factor positive, n (%)	7 (54)	11 (85)	8 (61)
Erosion on x-rays (%)	8 (61)	8 (61)	9 (69)
**Current Medications: **			
NSAIDs, n (%)	7 (54)	6 (46)	8 (61)
Methotrexate, n (%)	10 (77)	8 (61)	9 (69)
Hydroxycloroquine, n (%)	2 (15)	1 (7.9)	0 (0)
Sulphasalazine, n (%)	2 (15)	4 (31)	2 (15)
Leflunomide, n (%)	3 (23)	3 (23)	4 (31)
Prednisolone, n (%)	2 (15)	1(7.9)	1 (8)
Analgesics, n (%)	4 (31)	0 (0)	4 (31)

NSAID = non-steroidal anti-inflammatory drugs. P > 0.05 comparing all 3 groups in all the variables.

**Table 2:** Changes in the American College of Rheumatology (ACR) components and DAS 28 scores for the three groups of patients after 10 weeks

	**Electro-acupuncture (EA) (n = 12)**		**Traditional acupuncture (TCA) (n = 12)**		**Sham Acupuncture (n = 12)**	
	**Baseline**	**Week 10**	**Baseline**	**Week 10**	**Baseline**	**Week 10**
**Pain (VAS, 0–10)**	6.0 ± 2.1	5.7 ± 2.3	5.4 ± 2.8	5.1 ± 2.9	6.5 ± 2.0	5.1 ± 1.9
**Swollen Joints**	3.0 (0.3–6.0)	3.5 (1.3–4.0)	6.5 (4.3–9.8)	4.0 (2.3–6.8)	4.0 (2.0–7.8)	3.5 (1.3–5.0)
**Tender joints**	9.0 (3.0–13.5)	3.5 (2.0–9.0)*	13.0 (8.0–19.0)	9.0 (4.3–12.5)*	6.5 (4.0–17.3)	6.0 (2.0–12.5)
**Patient's Global (VAS, 0–10)**	6.4 ± 2.2	5.8 ± 2.1	6.4 ± 2.2	5.0 ± 2.4*	6.8 ± 2.3	5.0 ± 2.4*
**Physician's Global (VAS, 0–10)**	5.3 ± 2.1	4.0 ± 2.1*	6.0 ± 1.8	4.7 ± 2.1	5.1 ± 2.0	4.8 ± 2.3
**ESR (mm/hr)**	59 ± 33	58 ± 36	53 ± 26	59 ± 26	64 ± 41	66 ± 43
**CRP (mg/l)**	12.7 (5.6–46.7)	13.3 (5.6–38.2)	8.1 (4.3–24.4)	12.7 (5.1–43.1)	12.2 (5.0–43.6)	10.2 (4.9–36.2)
**HAQ**	1.5 ± 0.7	1.3 ± 0.7	1.6 ± 0.6	1.5 ± 0.7	1.4 ± 1.0	1.3 ± 0.9
**DAS28**	4.5 ± 1.0	4.4 ± 1.0	4.8 ± 1.2	4.7 ± 1.1	4.5 ± 1.3	4.4 ± 1.1

Data were expressed as mean ± SD or median (IQR). VAS = visual analog scale. ESR = erythrocyte sedimentation rate. CRP = C-reactive protein. HAQ = health assessment questionnaire. DAS 28 = disease activity score using the 28 joint counts. * p < 0.05 comparing before and after treatment.

### Dropout rate and compliance with the protocol

**Figure 3:**
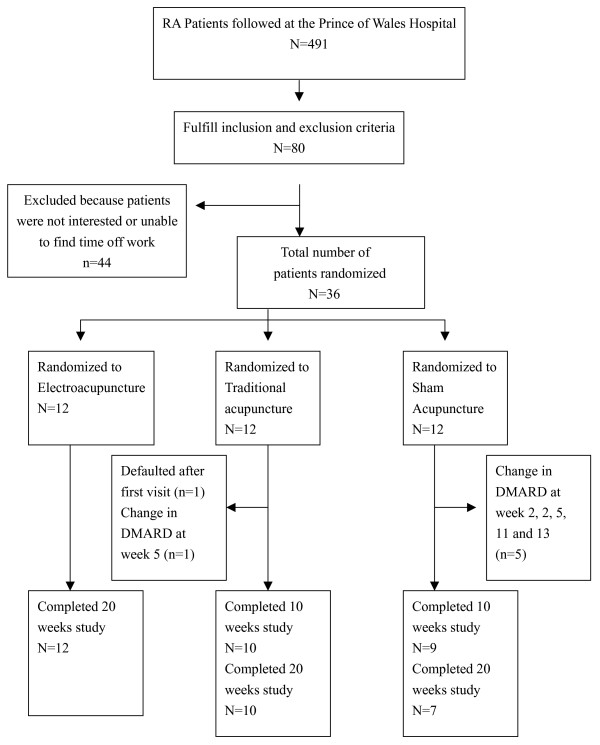
Patients progress through the trial.

Figure 3 summarized the patients' progress through the trial. Twelve, 10 and 7 patients from the EA, TCA and sham groups respectively completed the study at 20 weeks (p < 0.03); all except one of the premature dropouts were due to lack of efficacy requiring increase in the dosage of existing DMARD or addition of another DMARD. One patient from the TCA group defaulted follow up after the first visit. Another patient from the TCA group required change in DMARD at week 5. Five patients from the Sham group required change in DMARD at week 2, 2, 5, 11 and 13.

### Adverse events

Overall, the procedures were well tolerated; a total 3 episodes of minor adverse events were reported in 3 patients, including tingling sensation after acupuncture, herpes zoster and dyspepsia. The last 2 events were thought not to be related to the procedure.

### Variability of the primary clinical outcome measures and other preliminary outcome data

The variability of the primary clinical outcome in patients at baseline and at week 10, as shown by the standard deviation, is presented in Table 2.

There were no significant differences in the pain score at week 10 between the 3 groups (Table 2). However, when the different ACR core disease measures were analyzed separately at week 10, there was a significant reduction of the physician's global assessment score (p = 0.04) and the number of tender joints (p = 0.03) in the EA group. For the TCA group, significant reduction in patient's global assessment score and the number of tender joints was observed at week 10 (p = 0.01). All the ACR components remained unchanged except patient's global score improved in the Sham group (p = 0.03). There were no differences observed in the DAS28 scores between the 3 groups at any time point, or within the groups from baseline to week 10. There were no significant differences in the number of patients achieving ACR20 at week 10 between the 3 groups with 3 (25%), 2 (17%) and 2 (17%) patients in the EA, TCA and Sham groups respectively. The improvement in the ACR core disease measures were not sustained beyond week 10 in all 3 groups.

## Discussion

This pilot study found that about half of the RA patients with persistent active disease despite DMARDs were willing to try acupuncture as an adjunct for pain relief. The accrual rate was reasonable since this is only a single center study. Dropout rate was low for both electroacupuncture (0%) and traditional acupuncture (16.7%). However, the dropout rate for Sham acupuncture was rather high (25% at week 10, 41.7% at week 20). Adverse events were minimal.

This is the first reported randomized, controlled trial on the efficacy and tolerability of traditional Chinese acupuncture compared with electro-acupuncture and sham acupuncture in patients with refractory RA using multiple local and distal points. Although the pain scores did not differ between the 3 groups, this study shows that both traditional acupuncture and electro-acupuncture may serve as an adjunct by reducing the number of tender joints in patients with refractory RA compared to sham acupuncture. Previous studies published about the efficacy of acupuncture are difficult to interpret due to methodological problems [12]. The important point of our study is that it is a study in the methodology of acupuncture research as discussed in the following paragraphs.

Previous studies used different definitions for sham acupuncture, including insertion of needles adjacent to traditional point without manipulation [19], points with no known effects [10], points far away from the painful area without stimulation [20], distant arbitrary points [21], superficial insertion of needles into non-classical points [22], and real acupuncture point but no skin puncture [11]. However, some of these acupuncture points that were used may still be effective [21]. The retractable type of sham needle [23,24] has been intended for use in acupuncture-naïve subjects or subjects with limited acupuncture experience. However, in a Japanese study [25], 60% of the subjects could distinguish between sham and genuine needling, probably due to the fact that most people in Asian countries have more knowledge and experience with acupuncture. Our group found that sham sites and superficial punctures lead to inadequate blinding since more than 50–85% of recipients could immediately recognize that they are not receiving real acupuncture. The special device we used which hide the distal end of the needle was effective as 85% patients who received acupuncture in the hand and 90% patients who received acupuncture in the leg were unable to distinguish whether they were receiving real or sham acupuncture [15]. There was no significant difference between those who had received previous acupuncture or not [15]. In this study, there is no significant treatment effect demonstrated in the sham acupuncture group as all the outcome measures remained unchanged except the patient's global score. Moreover, significantly more patients from the sham acupuncture group dropped out prematurely due to inefficacy compared to the 2 acupuncture groups.

We chose these treatments to investigate a specific effect of the stimulation, or depth of needling (TCA or EA vs. sham acupuncture). We acknowledge the fact that waiting list controls may be useful to ascertain the extent of placebo effects of the acupuncture setting (for example, contact factors, talking and listening, and credibility of the intervention), patient preferences and expectation. Indeed, the fact that both the acupuncture and sham groups reported significant improvements in the patient's global score and a trend towards a reduction in the pain score suggests that acupuncture may elicit a greater placebo effect. However, the dropout rate for patients randomized to waiting list controls may be even higher than Sham acupuncture due to pre-randomization preferences for acupuncture. By having to wait for a treatment that they believe is effective, patients may also be disappointed by the delay, which may influence their ratings of subjective outcomes while waiting.

There have been 9 studies which examined the efficacy of acupuncture in RA [11,26]. Our study differs from others as 6 acupuncture points were used including local and distal points. Literature suggested that a combination of at least 4 distal and local points are required for treating systemic inflammatory diseases such as RA [27]. We have chosen the 6 acupuncture points since all of them can be regarded as distal points for the treatment of the underlying systemic inflammatory disease, as well as local points treating the arthritis of the nearby joints including the elbows, hands, wrists, knees and ankles based on the widely accepted Traditional Chinese Medicine theory of 'Bi' syndrome [17].

The acupuncture technique was questionable in previous studies as the duration of treatment were variable and the sensation of "de Qi" was not mentioned [10,11]. David et al employed only one single point selected for a short duration of 4 minutes *in situ *and manipulated at 2 minutes for 5 seconds [11], while Man and Baragar used electrostimulation of 3 well known classical points for 15 minutes [10]. Camerlian *et al *defined acupuncture as 20 minutes of electrostimulation of major classical points with "de Qi" [22]. According to both traditional Chinese and Western acupuncture theory, good results can be achieved when needles are left inserted for 15–30 minutes [13]. Therefore, we believed that a 40 minute – session with "de Qi" should be adequate for the proper treatment of our patients.

The lack of improvement in the pain score was disappointing. In general, analgesia is obtained by short-term acupuncture, whereas curative acupuncture requires long-term acupuncture treatment procedures. In the Man study, patients only received one single session of acupuncture [10] whereas in the David study, patients only received 5 weekly sessions. In standard practice, acupuncture treatment twice a week for at least 5 weeks may be required in chronic painful conditions like RA [13]. Hence, our study was carefully designed so that patients can receive 2 weekly sessions over a period of 10 weeks. We believed that this duration of treatment should be adequate in achieving positive results should there be any. However, since these patients are having advance disease, whether patients with earlier, more potentially reversible/treatable disease may benefit remains uncertain.

This is the first randomized study that demonstrated a reduction of the number of tender joints in patients with refractory RA after acupuncture, although the effect wears off soon after stopping acupuncture treatment. This study does not suggest any additional advantages of electroacupuncture over the traditional acupuncture. Electrical acupuncture might work best in situations that involved significant muscle spasm. Bhatt-Sanders has identified 5 positive studies which suggested acupuncture treatments give significant pain relief, 2 studies which showed no significant differences between real and placebo acupuncture [26]. However, these studies did not use the recognized outcome measures such as the ACR core components and the methodologies were problematic. Recent review has identified only 2 randomized controlled trials that provided the best evidence there is today. In the Man and Baragar study [10], a significant decrease in knee pain was reported with electroacupuncture at 24 hours, and 4 months post treatment. The outcome measure used is not currently recognized and the quality of the data was impacted by poor reporting and the use of hydrocortisone injection in the contralateral knee [12]. In the David study [11], acupuncture was shown not to be effective for those with symptoms of RA. The methodology was well described but the acupuncture technique was questionable. These studies illustrate the inherent difficulties related to acupuncture trials which included the design of the study, definition of acupuncture itself and elimination of bias as discussed before. Future study may consider incorporating the tender joint count as one of the main outcome assessment.

Five previous studies had examined acupuncture for potential anti-inflammatory effects [10,11,22,28], only one showed an anti-inflammatory effects with decreased rheumatoid factor, IgA, IgG levels, and decreased T cell activity with no change in ESR [29]. Our findings concurred with majority of the previous studies that no significant anti-inflammatory effect was observed in terms of changes in the DAS score, the number of patients achieving ACR20, ESR, CRP levels or other clinical parameters except a modest reduction of the number of swollen joints only in the group who received traditional Chinese acupuncture. Recent data suggests that acupuncture may have anti-inflammatory action via release of neuropeptides e.g. calcitonine gene-related peptide from nerve endings [30]. Whether this is also true in RA would need to be addressed in future trials as one of the secondary outcomes.

This is a study of the methodology of acupuncture research and thus confined by a clear-cut regimen. This is very different from the usual traditional Chinese acupuncture practiced which is characterized by a holistic approach to the management of the disease. Therefore, the practitioner of traditional acupuncture will approach each patient with a personalized treatment plan rather than treating all patients with RA using a standard protocol. In practice, practitioners tend to use certain patterns of points on all of most of their patients [31]. Our treatment protocol was specially designed to reflect the best practice based on the widely accepted Traditional Chinese Medicine theory of 'Bi' syndrome as this could be replicable.

## Conclusion

Our preliminary evidence suggests that electroacupuncture and traditional acupuncture were no better than sham acupuncture in terms of changes in the pain score. Acupuncture is safe and may be effective as an adjunct in reducing the number of tender joints, although no clear anti-inflammatory effects had been demonstrated. Further preliminary studies may consider including both pain score and tender joint count as the main outcome assessment. This pilot study has allowed a number of recommendations to be made to facilitate the design of a large-scale trial, which in turn will help to clarify the existing evidence base on acupuncture for RA.

## Competing interests

The author(s) declare that they have no competing interests.

## Authors' contributions

LST is responsible for the conception and design of the trial, clinical assessment, analysis and interpretation of data; and drafting the manuscript.

TKL is responsible for the acquisition of data.

LZ is responsible for the design of the trial and performing acupuncture on all the patients

PCL is responsible for the conception and design of the trial and revising the manuscript critically for important intellectual content.

EKL is responsible for the conception and design of the trial, clinical assessment, drafting the manuscript and revising it critically for important intellectual content.

All authors read and approved the final manuscript.

## Pre-publication history

The pre-publication history for this paper can be accessed here:


